# Correlation of cardiac output measured by non-invasive continuous cardiac output monitoring (NICOM) and thermodilution in patients undergoing off-pump coronary artery bypass surgery

**DOI:** 10.1007/s00540-014-1938-z

**Published:** 2014-11-08

**Authors:** Hoiyin Cheung, Quan Dong, Rong Dong, Buwei Yu

**Affiliations:** Department of Anesthesiology, Ruijin Hospital, Shanghai JiaoTong University School of Medicine, 197 Ruijin Er Road, Shanghai, 200025 People’s Republic of China

**Keywords:** Measurement techniques, Cardiac output, Thermodilution, Non-invasiveness, Bioreactance

## Abstract

**Purpose:**

This observational study was designed to evaluate the clinical value of cardiac output (CO) obtained via bioreactance (NICOM™) as compared with values of CO obtained via thermodilution (using pulmonary artery catheter, Vigilance™) and the thoracic bioimpedance (BioZ.com™), in patients undergoing off-pump coronary artery bypass surgery.

**Methods:**

Fifty American Society of Anesthesiologists physical status I–III patients, aged 38–81 years, scheduled for off-pump coronary artery bypass surgery were enrolled in this study. CO data (NCO, BCO, PCO) were recorded during the operative period at ten time points after stable hemodynamic conditions were achieved.

**Results:**

The equation of the relationship between the PCO and NCO is PCO = 0.945 × NCO + 0.328 (*r* = 0.77), and that of PCO and BCO is PCO = 0.965 × BCO + 0.729 (*r* = 0.63). Furthermore, no statistical difference was found between PCO versus NCO (mean (SD): 4.4 (1.1) versus 4.4 (0.9), *p* = 0.431). A significant correlation was found between PCO and NCO (*r* = 0.77, *p* < 0.001). Correlation was also found between PCO and BCO (*r* = 0.63, *p* < 0.001).

**Conclusions:**

The NICOM device is a safe, convenient, and reliable device for measuring continuous non-invasive cardiac output and cardiac index, and the trends of change in CO during the surgery are similar between NICOM and PAC.

## Introduction

Cardiac output and cardiac index are key parameters of hemodynamics and can be used to evaluate cardiac pump function and calculate other parameters like SVR or PVR and so on. In patients undergoing cardiac surgery, thermodilution using a pulmonary artery catheter (PAC) is still the reference gold standard. However, although the incidence of complications with the PAC is relatively low, the technique is still quite invasive and up to now, there is no clear evidence of improved outcomes associated with its insertion and use to guide therapy [1–5]. Therefore, several less-invasive methods have been proposed [6, 7]. Non-invasive methods could be preferable, especially for low-risk patients in whom CO monitoring is increasingly used nowadays, as non-invasive methods have fewer side effects as compared to invasive procedures. One of these non-invasive methods is thoracic bioimpedance, which analyzes intrabeat variations of transthoracic voltage in response to an input high-frequency current; however, inconsistent measurements were found in intensive care [8, 9, 16].

Recently, a new signal-processing method has been developed which is called NICOM. Its signal is based on the frequency modulation and phase modulation of the output voltage. This advanced bioreactance technology improves the signal-to-noise ratio 100-fold. However, the accuracy of NICOM has not been confirmed, especially when compared with the PAC technique in patients undergoing off-pump coronary artery bypass surgery. Therefore, we conducted the following investigation.

The aim of this study was to evaluate the accuracy of CO measured by NICOM in patients undergoing off-pump coronary artery bypass surgery, and to compare the results with the other two devices: (1) the BioZ.com™ system with thoracic bioimpedance, and (2) continuous CO monitoring using a PAC with thermodilution.

## Patients and methods

Patients undergoing off-pump coronary artery bypass surgery gave their informed consent to the study, which was approved by the Ethics Committee of Shanghai Ruijin Hospital (2013/NO.69). Inclusion criteria were ASA I–III or NYHA I–II. A total of 50 patients were recruited for the current study with the insertion of PAC. When the patients arrived in the operating room, they were connected to patient with the NICOM and the BioZ.com placed on the each side of the thorax. The CO data and standard hemodynamic data (HR, MAP, SV, and SVI) from all devices were record by an observer who was not responsible for the patient’s anesthesia. The measurements were free of interference from surgery or infusion boluses (such as Ringer’s solution and succinylated gelatin or vasoactive agents). Predefined measurement points were: T1 = immediate after induction of anesthesia (study initiation), T2 = 5 min post-induction, T3 = 10 min post-induction, T4 = sternotomy, T5 = 5 min post-sternotomy, T6 = 10 min post-sternotomy, T7 = opening of pericardial, T8 = pericardial closure, T9 = sternal closure, T10 = 5 min post-sternal closure.

## Statistics

Data are expressed as mean (SD) or median (range) for non-normally distributed variables or number and percentage as appropriate. CO values obtained from NICOM and PAC or BioZ.com and PAC were also using ANOVA. The correlation of CO was determined by linear regression. The Bland–Altman analysis was used to compare bias and limits of agreement.

A *p* value of 0.05 was considered as statistically significant and all *p* values were two-tailed. Statistical analysis was done by using SPSS for Windows Release 20.0 (SPSS Inc., Chicago, IL, USA).

## Results

**Fig. 1:**
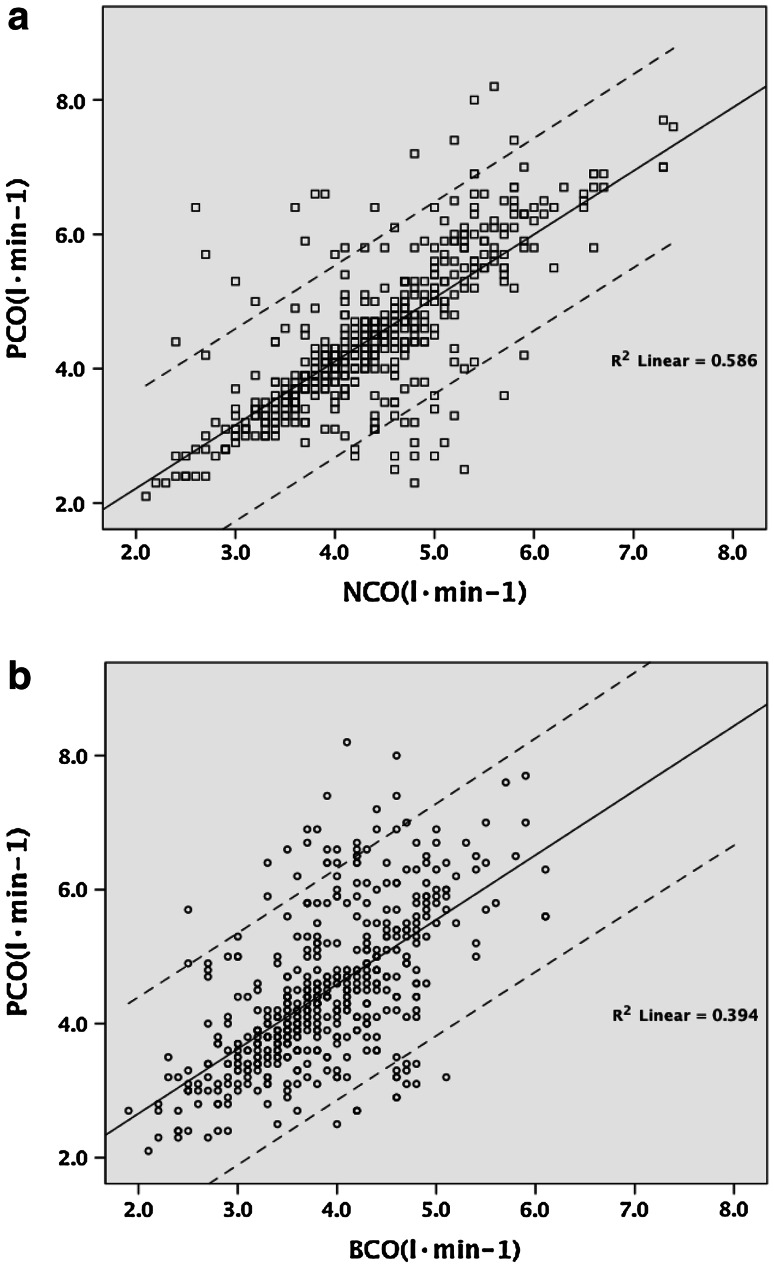
Relationship between PCO and NCO (**a**) and PCO and BCO (**b**). *CO* cardiac output, *NCO* cardiac output by NICOM, *PCO* cardiac output by PAC, *BCO* cardiac output by BioZ.com. The *continuous line* indicates the regression line. The *dashed lines* indicate 95 % confidence intervals

**Fig. 2:**
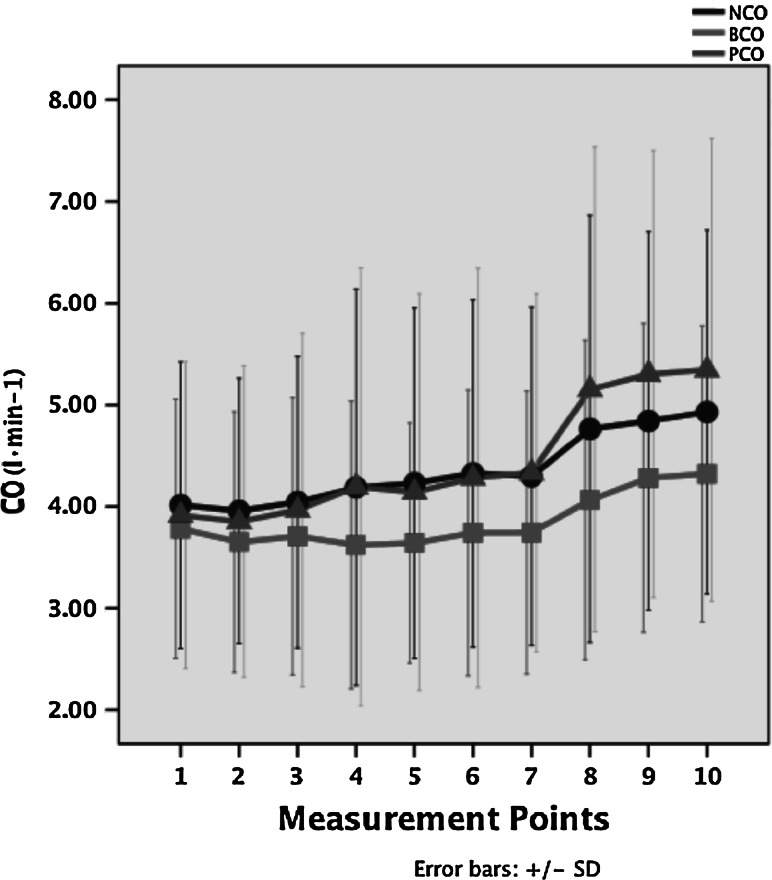
CO values trend of changes during the surgery. *CO* cardiac output, *NCO* cardiac output by NICOM, *BCO* cardiac output by BioZ.com, *PCO* cardiac output by PAC. The* horizontal axis* represents measurement points

**Fig. 3:**
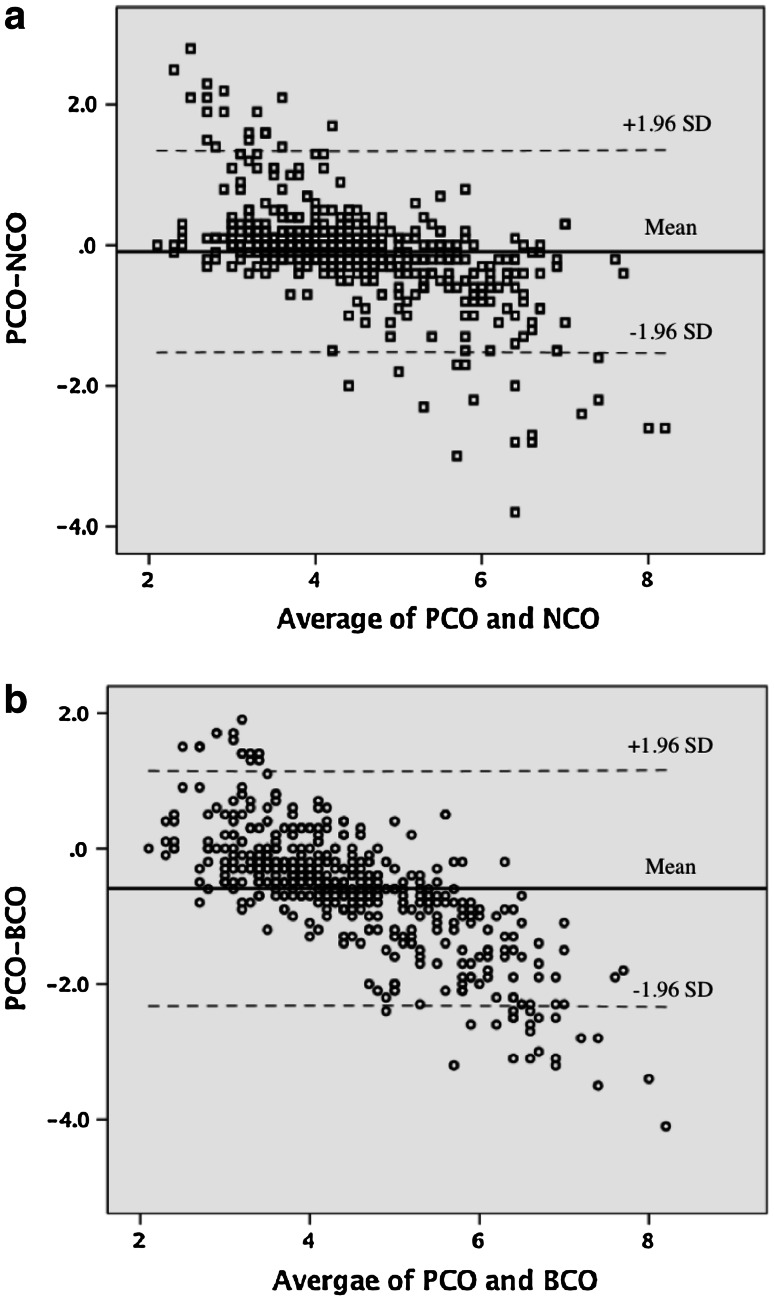
Bland–Altman analysis between **a** PCO and NCO, and **b** PCO and BCO. *NCO* cardiac output by NICOM, *BCO* cardiac output by BioZ.com, *PCO* cardiac output by PAC

Baseline characteristics for the 50 subjects are indicated in Table 1. No statistical difference was found between PCO versus NCO (*p* = 0.431) (Table 2). Figure 1a shows the relationship of CO data between PAC and NICOM; the equation of the relationship is PCO = 0.945 × NCO + 0.328 (*r* = 0.77). Figure 1b shows the relationship of CO data between PAC and Bioz.com; the equation of the relationship is PCO = 0.965 × BCO + 0.729 (*r* = 0.63). Figure 2 shows the variations in CO for PAC, NICOM and BioZ.com in different measurement points during the surgery. PCO and NCO increase at a slower rate from T1 to T7 but increase at a faster rate from T8 to T10, whereas BCO increases gradually from T1 to T10. The Bland–Altman analysis was used to compare the bias, precision (SD of bias), and limits of agreement [bias (1.96 SD)]. The bias and limits of agreement of CO between PAC and NICOM are depicted in Fig. 3a and the bias and limits of agreement of CO between PAC and BioZ.com are depicted in Fig. 3b.

**Table 1:** Baseline characteristics of patients (*n* = 50)

Sex (M/F)	38/12
Age (year)	65 (38–81)
Height (cm)	166 (154–181)
Weight (kg)	69 (49–86)
Hypertension	29 (58)

Values are median (range) or number (%)

**Table 2:** Comparison of cardiac output (*n* = 50) between NICOM and PAC and between BioZ.com and PAC

	NICOM	BioZ.com	PAC
CO (l/min)	4.4 (0.9)	3.9 (0.7)	4.4 (1.1)
*p* value*	0.431	<0.001	

Values are mean (SD)

*CO* cardiac output

* Compared with PAC

## Discussion

Our study shows that NICOM is convenient and safe in a clinical setting, as it only requires the placement of two double electrodes on the patient’s thorax. Correlation coefficient of PCO and NCO (*r* = 0.77) is better than those of PCO with BCO (*r* = 0.63). Although NICOM up to now cannot replace the use of PAC, it provides consistent continuous non-invasive measurements of CO and CI and seems to be able to track in real time the trends and the change with the time. There is literature that suggests that real-time tracking of the trends of change in CO could be more important than the ability of the monitor to deliver a highly accurate single measurement under stable conditions [10]. In the current study, the trends of change in CO during the surgery are similar among the three devices, and the trends by NICOM are closer to PAC than by BioZ.com (Fig. 2). From Fig. 3a we can deduce that for values of CO between 4 and 6 NICOM and PAC values have a good correlation. For values of CO below 4, NICOM overestimates values of CO as compared to PAC. For values of cardiac output above 6, NICOM underestimates values of CO as compared to PAC. Similarly, from Fig. 3b we can deduce that for values of CO between 4 and 6, BioZ.com and PAC values have a good correlation. For values of CO below 4, BioZ.com overestimates values of CO as compared to PAC. For values of CO above 6, BioZ.com underestimates values of CO as compared to PAC.

As an invasive hemodynamic monitor, PAC was regarded as a gold standard of reference because we have no better reference for continuous CO monitoring [11–14]. We therefore compared the relationship between non-invasive cardiac output monitor (NICOM) with PAC. In the current study, we are still not sure if NICOM can replace the PAC as a means to measure CO. A meta-analysis study literature also showed that none of the four tested alternative methods (pulse contour analysis, esophageal Doppler, thoracic electrical bioimpedance) achieved agreement with PAC [15]. However, considering that the scope of PAC’s applicability is limited, and that it also brings a lot of risks and complications (e.g., infection) [20], the clinical hemodynamic monitoring is moving towards less invasive or even non-invasive methods.

Bioimpedance is based on the fact that the conductivity of a high-frequency, low-magnitude alternating current passed through the thorax changes as blood flow varies with each cardiac cycle. These changes can be measured using electrodes placed on a patient’s chest and used to generate a waveform from which cardiac output can be calculated. However, its accuracy is always in doubt, as the technique has its own limitation [16].

Bioreactance is developed out of bioimpedance and measures changes in the frequency of the electrical currents traversing the chest, rather than changes in impedance, potentially making it less sensitive to noise [17, 18]. Also, bioreactance-based NICOM has acceptable accuracy in challenging clinical environments [19]. Furthermore, one study suggests that NICOM can be clinically valid when used in ICU patients [21, 22], but the accuracy of NICOM and Bioz.com have not been confirmed, especially compared with the PAC technique in patients undergoing off-pump coronary artery bypass surgery. This is why we compare the three devices to evaluate the accuracy of CO measurements in patients undergoing off-pump coronary artery bypass surgery. The measurement points in this study were defined according to clinical importance of surgical step, as they are also very common in cardiac surgery.

The limitations of the current study are as follows: Firstly, all the data for the patient group were collected after induction of anesthesia. PAC values before anesthesia are unavailable, because we abstained from placing a PAC in the patient who is awake so as not to further increase discomfort before surgery. Secondly, the study was limited only to patients undergoing cardiac surgery and did not take into account post-surgical patients in the ICU. Thirdly, the current study did not evaluate the ability of the NICOM to predict fluid responsiveness.

In conclusion, the NICOM is a safe, convenient device, and reliable to continuously and non-invasively monitoring cardiac output and cardiac index, and could track the trend of changes under dynamic conditions. Although the device cannot replace PAC for patients undergoing off-pump coronary artery bypass surgery at present stage, it may potentially be used in a wide range such as during laparoscopic surgery or for the patients with heart diseases undergoing non-cardiac surgery, when invasive monitoring is not an option or not desired.
